# Digital Detection of DNA via Impedimetric Tracking of Probe Nanoparticles

**DOI:** 10.1021/acs.nanolett.4c05324

**Published:** 2025-04-22

**Authors:** Mohammad Saghafi, Suryasnata Tripathy, Taghi Moazzenzade, Jurriaan Huskens, Serge G. Lemay

**Affiliations:** † Department of Molecules and Materials, Faculty of Science and Technology, 3230 University of Twente, 7500 AE Enschede, The Netherlands

**Keywords:** electrochemical impedance spectroscopy, nanoelectrode, stochastic biosensor, toehold-mediated DNA displacement, sensor array, high frequency

## Abstract

CMOS-based nanocapacitor arrays are an emerging technology that permits spatially resolved, high-frequency impedance measurements at the nanoscale. Their capability to detect micro- and nanoscale entities has already been established through nonspecific interactions with the targets. Here, we demonstrate their application in specific macromolecular capture and detection using single-stranded DNA (ssDNA) as a model analyte. While individual ssDNA strands fall below the detection threshold, we employ a strand displacement assay that links DNA hybridization to target ssDNA induced displacement of reporter nanoparticles. This displacement reaction results in distinct electrical signatures with complex spatiotemporal patterns, details that remain unresolved in conventional macroscale impedance spectroscopy techniques due to their limited resolution and signal averaging that obscures localized interactions. The proposed system’s massively parallel architecture and the ability to detect complex dynamics of individual nanoparticle–nanoelectrode interactions make it a promising candidate for scalable, portable, and cost-effective biosensing applications in clinical diagnostics and beyond.

## Graphical abstract

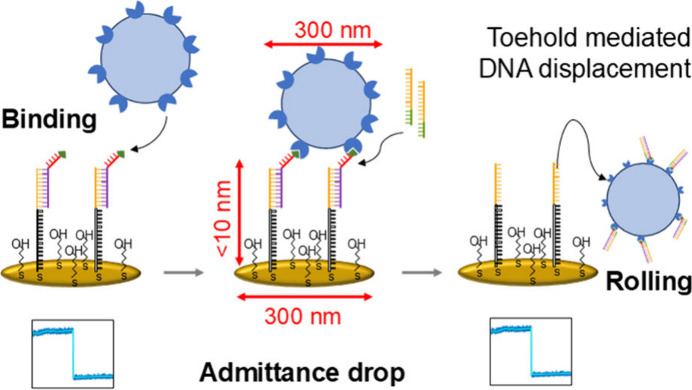

Development of detection modalities for proteins and nucleic acids in trace amounts is greatly desirable given their many applications in fundamental biological studies, gene therapy, clinical diagnostics, disease prognosis monitoring, and drug discovery. Over the past decade, several strategies have been developed and experimentally validated, which has helped to push the detection level to subfemtomolar concentrations. Most of these approaches have been based on optical transduction principles, whether fluorescently labeled immunosorbent assays^1,2^ or other nonfluorescent schemes such as surface-enhanced Raman scattering,^3^ surface plasmon resonance microscopy,^4^ and plasmonic nanocavities.^5^ For instance, the digital enzyme-linked immunosorbent assay (ELISA) platform reported by Rissin et al.^1^ detects as few as 10–20 enzyme-labeled complexes in 100 μL of sample (∼10^–19^ M) using arrays of femtoliter-sized reaction chambers, while Cohen et al.’s droplet digital ELISA platform^2^ accounts for a limiting detection of 20 aM, corresponding to ∼1200 protein molecules. A well composed review of optical single molecule detection schemes was provided by Zijlstra and coauthors,^6^ and a tutorial review by Holzmeister et al.^7^ discusses in detail the associated high and low concentration limits.

Nonetheless, despite their many advantages, optical schemes often pose practical limitations due to complex experimental protocols, bulky and expensive instrumentation, and the need for skilled operation. Therefore, alternative detection modalities were also explored in the past decade, primarily toward addressing several of these challenges: (i) ensuring relative simplicity, (ii) cost-effectiveness, (iii) scalability, and (iv) portability for clinical diagnosis.^8^ Particularly, for the detection of nucleic acids in trace amounts, biosensing platforms relying on electrical transduction have been considered promising candidates.^9^ While some of the electrical approaches utilized nanomaterials,^10^ others relied on direct interactions with DNA and RNA.^11^ Recently, an ELISA-like electrolyte-gated organic transistor array-based prototype has been reported, which is capable of simultaneous single protein and single DNA detection.^12^

Among the different strategies employed for ultrasensitive nucleic acid detection, toehold-mediated strand displacement (TMSD) is of particular interest given its significance in DNA nanotechnology,^13^ sensing and therapeutics,^14^ computing,^15^ and clinical diagnosis.^16^ Although optical characterization of DNA binding and strand displacement has been demonstrated with single molecule fluorescent assays on DNA origami microarrays,^17^ electrical detection of strand displacement with such sensitivity remains elusive.^18^ Specifically, the development of all-electrical strand-displacement sensors for clinical diagnosis with picomolar or lower limits of detection still poses several fundamental challenges, including the development of suitable sensing platforms, mass transport, and reaction kinetics. At such limiting concentrations, resorting to stochastic approaches provides a viable means for analyte capture, accurate interpretation of experimental findings, and associated decision making.^19^

The present proof-of-concept study, realized using complementary metal-oxide semiconductor (CMOS) nanocapacitor arrays, represents a major step in that direction. In this exploratory work, we successfully captured the complex spatiotemporal dynamics of individual nanoparticle–nanoelectrode interactions in real-time as a means to monitor the TMSD phenomenon. Notably, the ability to probe these interactions with accuracy and precision could have significant scientific implications such as enabling real-time observation of nanoscale molecular kinetics and facilitating precise detection of low-abundance targets in complex biological fluids. In this context, the nanocapacitor array’s potential to distinguish such interactions from the background noise, while preserving the underlying dynamics, represents a key scientific advancement. However, this ability to precisely track individual particle binding and unbinding events on a nanoelectrode as well as probing localized conformational changes necessitates further refinement to allow DNA detection at trace concentrations, as elucidated further below.

We employ electrode arrays fabricated using 90 nm CMOS technology and comprised of 65,536 individually addressable nanoelectrodes (∼300 nm diameter) arranged in a 256 × 256 matrix with ∼0.6 × 0.8 μm pitch (Figure S1).^20^ A schematic view of a 3 × 3 subset of electrodes and corresponding atomic force microscopy (AFM) data are shown in Figure 1a and Figure 1b, respectively. This fully electronic platform can perform high-frequency measurements up to 70 megahertz (MHz), thereby circumventing the Debye screening limit over a wide range of electrolyte salt concentrations.^20,21^ Due to the switching mode of operation of the array, phase information is not collected; we therefore report the amplitude of the effective admittance for the electrode–electrolyte system.^22^ The limit of detection of single-electrode measurements lies in the range of 0.1–0.3 nanosiemens (nS) at 50 MHz. Each row of electrodes takes turns serving as a working electrode, while the remaining rows function as the counter electrode. The electrodes are read columnwise, which allows addressing each electrode separately. Under physiological salt concentrations, probing at frequencies below 5 MHz is sensitive to the electrochemical double layer near the electrode’s surface. At frequencies above 5 MHz, the measurement instead targets the conductance of the electrolyte in the surrounding volume. When a nanoparticle blocks an electrode at these higher frequencies, it causes a drop in admittance due to the depletion of ions and the consequent drop in conductance of the medium around the electrode. Unblocking the electrode has the opposite effect.^20,21,23^ Using these arrays, we have previously demonstrated real-time imaging of microscale particles,^23^ microemulsions,^24^ and living cells,^25^ as well as the ability to detect individual nanoparticles.^20^ Earlier, Widdershoven et al.^26^ had also demonstrated the detection of insulating paramagnetic beads (diameter 300 nm), capturing individual collision events as well as Brownian motion driven diffusion of bead clusters, while using an open dual in-line package for chip mounting. These early experiments, though carried out without analyte-specific surface modification, enabled further advancements in digital sensing at physiological salt concentrations.

**Figure 1:**
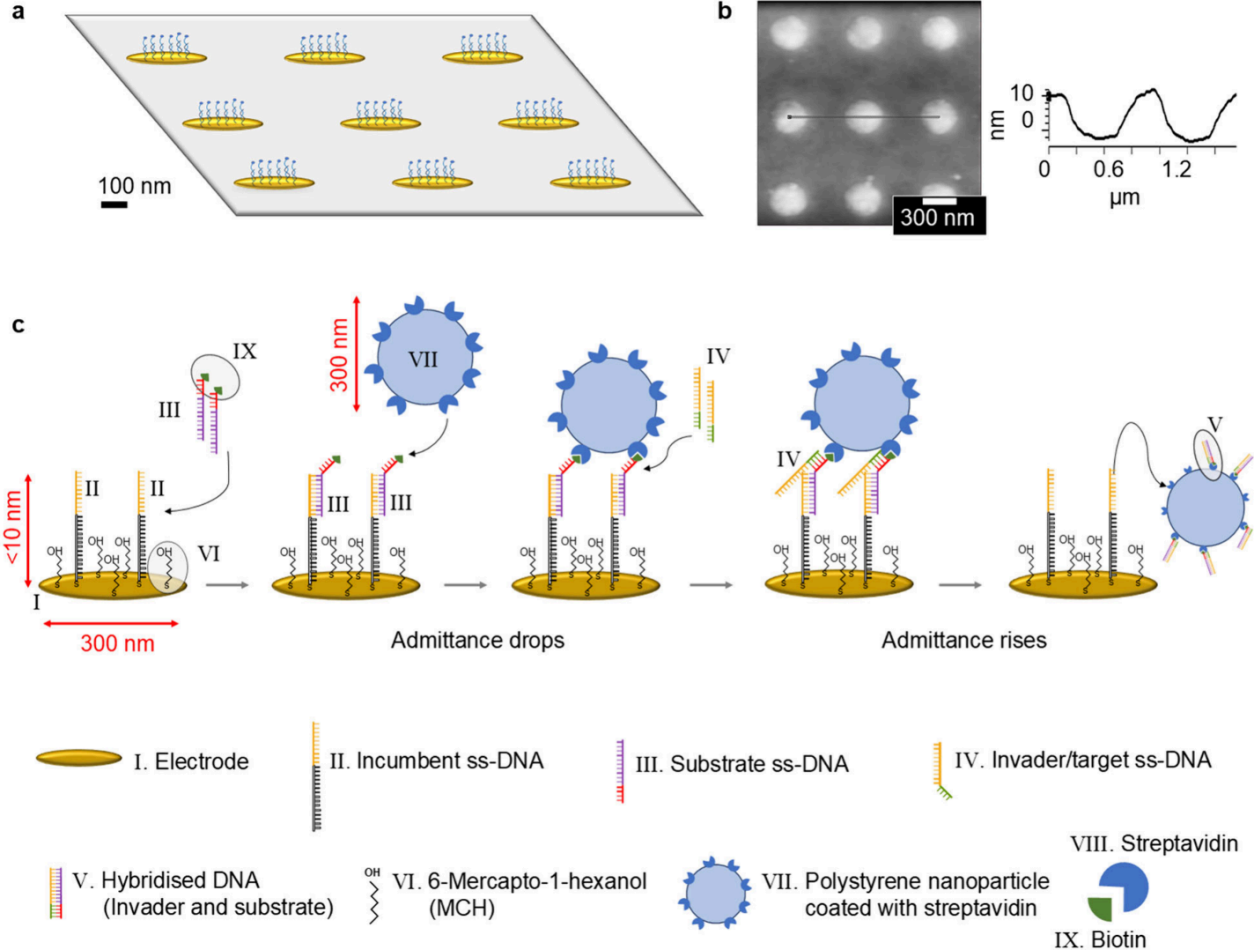
Simplified principle of operation of toehold-mediated strand displacement amplified by nanoparticles and monitored by an impedimetric nanoelectrode. (a) Schematic of a 3 × 3 subset of the 256 × 256 nanoelectrode array, in which each electrode (I) is an individually addressable impedimetric sensor. (b) (left) Corresponding AFM height image. (right) Line scan along the black line in the height image indicating that the electrodes have a diameter of ∼300 nm and a height of ∼10 nm. (c) Schematic representation of the toehold-mediated strand-displacement-based assay on the sensor. A nanoelectrode (I) functionalized with incumbent DNA (II) and MCH (VI) is exposed to biotinylated substrate DNA (III), allowing the binding of SAV-PS particles (VII) with sizes comparable to the electrode. The target DNA (IV) binds to the toehold region of the substrate, displacing the incumbent and releasing the hybridized substrate–target strands (V) along with the attached particle. The particle may leave or nonspecifically rebind to the electrode, causing rises or drops in admittance recorded by the nanoelectrode (an example is shown in Figure S7). The depiction of two anchors per particle in the figure is a conceptual representation. In reality, based on previous studies, a 300 nm particle can have up to 66 anchors.^27^

The TMSD-based assay is depicted in Figure 1c for a representative single nanoelectrode from the array (an illustration focusing solely on the DNA component is also provided in Figure S2). The individual nanoelectrodes (I) are first functionalized with a thiol-modified ssDNA (II) termed the incumbent. Subsequently, the rest of the electrode surfaces are functionalized with 6-mercaptohexanol (MCH), a thiol-modified short carbon chain with negatively charged heads (VI). While the incumbent acts as receptor for further functionalization, MCH provides antifouling functionality, limiting nonspecific binding.^28^ The incumbent is then hybridized with another ssDNA, termed the substrate (III). The substrate consists of a sequence complementary to the incumbent ssDNA but also containing a toehold that remains unbound during hybridization with the incumbent. The substrate is biotinylated at its 5′ end, which allows it to be tagged with a nanoparticle (VII). In this study, streptavidin coated polystyrene particles (SAV-PS), with diameters of 300 and 800 nm, were used for this purpose. The target (IV) can bind to the toehold part of the substrate and compete with the incumbent until it fully displaces it, releasing the substrate, target, and attached particle.^29^ The particle may either be carried by the flow or nonspecifically rebind to the electrode.

As shown in Figure 1c, the substrate (III) hybridizes with the incumbent (I) and is subsequently displaced by the target (IV). However, because the electrode is coated with a dense layer of MCH and the substrate molecules are small, observing these events without a particle probe is challenging. This limitation is particularly pronounced when the surface concentration of incumbents is intentionally kept low, as in our case, to reduce the number of anchors holding the particle, thereby facilitating the displacement process.

This specific protocol has previously been validated in our laboratory on macroscopic surfaces using a quartz crystal microbalance (QCM).^27^ In this work, the binding of a particle to a nanoelectrode and its subsequent dynamics were instead detected via real-time AC measurements at 50 MHz. Contrary to the QCM method, which is sensitive to the average surface coverage, we locally resolve individual particles impinging on the array. The particle size being comparable to the electrode size, this sensing becomes highly discrete, since an electrode can only accommodate one particle. Although this strategy is ultimately aimed at direct digital detection of strand displacements, the present article focuses on particle detection and tracking as indicators of the said displacement phenomenon. While this work only serves as a qualitative proof of concept, it lays the groundwork for developing an all-electronic platform for detecting trace amounts of DNA (near or at single molecular resolution) in the future, contingent upon further optimization of the experimental conditions. To that end, an important requisite would be to attribute individual particle displacements to single- (or a few) strand-displacement events, which will in turn require the hosted particles to be tethered to a single (or a few) anchor(s).

The complete surface functionalization process was monitored in real time at frequencies ranging from 1.6 to 50 MHz, which allowed observing changes in both the electrical double layer capacitance and the medium conductance around the electrode caused by the various functionalization steps (Section S5). The process included electrode cleaning, functionalization with the thiol-modified incumbent and MCH, and hybridization with the biotinylated substrate. Monitoring self-assembled monolayer formation using this platform has been reported previously.^30^

The data recorded by individually addressable electrodes during surface functionalization allow for the detection and exclusion of malfunctioning electrodes, as well as those with incomplete functionalization. This detection is based on the assumption that under identical medium conditions the recorded signals should follow a Gaussian distribution. The ability to statistically identify and exclude invalid electrodes from the data analysis is a key advantage of our parallelized approach compared to conventional sensors, which produce responses averaged over a larger area (Section S6).

Following surface functionalization with incumbent DNA, MCH, and substrate DNA, the electrodes were exposed to SAV-PS particles to create ‘host’ electrodes. Both the electrode size and the pitch of the array are comparable to the size of the SAV-PS particles themselves. Thus, while the binding of a single nanoparticle typically does not alter the average signal over the whole array significantly (Figure S7), each individual electrode may exhibit a substantial signal change when a particle binds to or unbinds from that electrode. Neighboring electrodes may also exhibit a response when a particle binds, albeit a smaller one. Figure 2a shows how the capture of a PS particle on an individual nanoelectrode is characterized by a sharp negative drop (negative step) in the admittance signal (electrode E1, top trace). This is caused primarily by an increase in solution resistance due to the excluded electrolyte volume near the electrode. Notably, these pronounced signatures are clearly distinguishable despite signal drift and high-frequency noise due to the clear separation in the time and amplitude scales involved. The middle and bottom traces in Figure 2a correspond to simultaneously performed measurements at neighboring electrodes E2 and E3. Here some smaller steps are observed that occur simultaneously at two neighboring electrodes, as indicated by the vertical arrows. We interpret this as being caused by a particle landing near the edge of an electrode, such that it influences the signal in two channels. In such cases, we consider the largest step as the primary event. We have never observed an event with such a simultaneous signature at electrodes that are not immediate neighbors (see also Section S8.2). Occasionally, rapid oscillations are also observed between two electrodes, as can be seen here for E2 and E3. Such oscillations were observed between electrodes in adjacent rows as well as columns.

**Figure 2:**
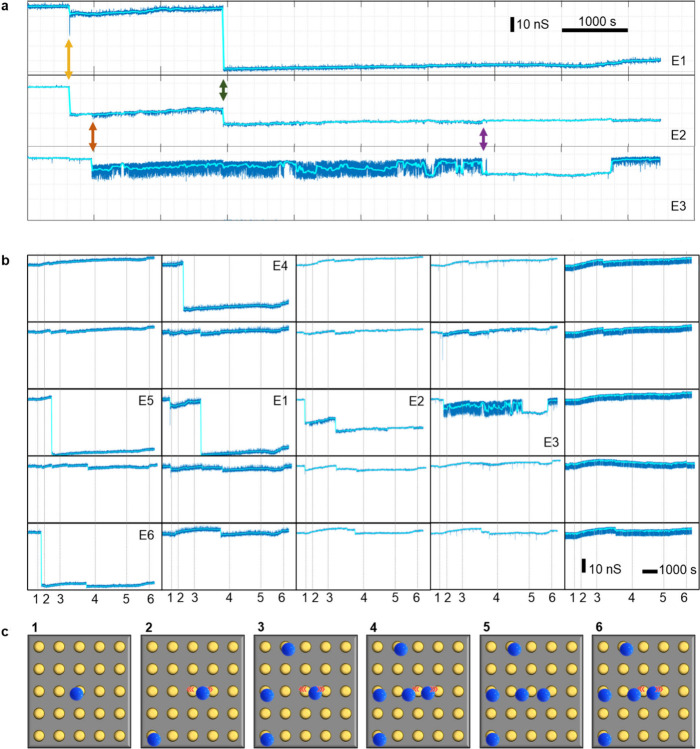
Particle dynamics tracking. (a) Admittance signals recorded at 50 MHz from three adjacent electrodes. E1 exhibits a particle binding, E2 shows steps smaller than the primary binding signature, and E3 displays a particle undergoing oscillations toward E2. The dark blue lines represent the raw signals, while the cyan lines are the denoised data (as described in Section S6). Since different (columns of) electrodes exhibit slight differences in signal amplitude, the curves have been shifted vertically for clarity. (b) Simultaneously recorded signals for 25 neighboring electrodes (5 × 5 grid). (c) Illustration of six inferred configurations of particles on the 25 neighboring electrodes at the six moments corresponding to vertical gray lines in (b). The arrangement of the electrodes in these sketches corresponds to the positions of the graphs in (b).

Figure 2b shows the simultaneously recorded signals at six neighboring electrodes arranged on a 5 × 5 grid. Six electrodes, labeled E1–E6, exhibit particle-induced signatures (E1, E2, and E3 are the same traces as in Figure 2a). Electrodes E4, E5, and E6 bind particles with only minor influence on their neighbors, indicating that the particles are captured near the center of the electrodes. In contrast, the other two particles are captured near the electrode edges, leading to crosstalk between electrodes E1, E2, and E3. Based on the position and timing of the steps, the positions of the particles can be deduced. Figure 2c shows six inferred configurations of particles on the 25 neighboring electrodes at the six times corresponding to vertical gray lines in Figure 2b (labeled 1 to 6). A variety of behavior is observed. A particle is bound to the left edge of electrode E2, whose signature is detectable on electrode E1 (yellow arrow). The same particle later rolls toward E3, exhibiting significant oscillatory behavior in E3 and minor oscillations in E2 (red arrow), before it eventually stops moving at a position closer to E3 compared to its initial position. This change in position is further detectable through the negative step on E3 and the simultaneous positive step on E2 (purple arrow). E1 receives a particle on its right edge, sharing its signature with electrode E2 (green arrow).

Given the large amounts of data generated during each experiment, the process of identifying the steps was automated. The methods for signal denoising and step detection are detailed in Section S6. The data shown in the main text are from one representative experiment for the sake of consistency.

Figure 3a shows the results of analyzing the signals from all valid electrodes and extracting the time and size of each step during this typical experiment. Each point in this amplitude–time scatter plot represents a single detected step. At early times (first shaded region) no particles were present, and the distribution was characterized by small steps (typically <2 nS) caused by random noise. At 500 s, particles began to impinge upon the array. At this point the size of the steps around the baseline increased, and simultaneously a new type of events characterized by large negative steps (predominantly in the range −20 to −50 nS) appeared. The rate of occurrence of these large events diminished drastically over the scale of ∼30 min. Figure 3b shows a map of the spatial location of all steps more negative than −15 nS. They are homogeneously scattered over the array (apart from some small areas and columns corresponding to electrodes that were eliminated during preanalysis). The distribution is not entirely random, however: once an event has occurred at an electrode, the chance of a subsequent event at one of the eight neighboring electrodes becomes essentially zero. Upon replacing the particle solution with phosphate saline buffer (PBS) solution (around 7500 s), the frequency of the large events decreased further, while the fluctuations around the baseline (small steps) remained largely unaffected. Finally, as soon as a solution containing the target solution was introduced (around 8200 s), a large number of large negative steps were once again observed. Throughout the process, large positive steps were extremely rare.

**Figure 3:**
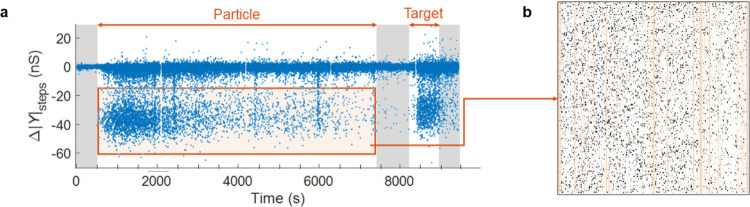
(a) The amplitude of all detected steps during an experiment as a function of time; gray sections indicate PBS exposure as control phases. (b) The map of electrodes at which steps more negative than −15 nS occurred. The image consists of 256 × 256 pixels, each representing one electrode. Electrodes in black had recorded large steps, while electrodes in pale peach were tagged as invalid. The vertical pattern of excluded electrodes arises from the fact that each column of electrodes is connected to a single current readout circuit on the chip. A malfunction or outlier output in that circuit invalidates the entire column of electrodes in our analysis. The flow direction is vertical in this image.

The large negative steps during the particle binding step can be attributed to the binding of NPs to the electrodes. The decrease in binding rate with time indicates that the eligible electrodes are becoming saturated, consistent with the observed exclusion at the neighboring electrodes. This behavior can be caused by the rearrangement of flow around bound particles, enhanced by electrostatic interactions between particles. The increase in small steps during the particle binding phase can be attributed to the passage of particles that do not bind and particle rearrangements, as discussed further below. The decrease in the rate of large events upon introducing PBS solution is consistent with this interpretation, with the caveat that some particles may have remained adsorbed in the fluidic interconnects, leading to a few rare events.

The dramatic resurgence of negative steps upon introducing the target is, however, *a priori* unexpected. TMSD would be expected to lead to the release of particles and hence to large *positive* steps complementary to the corresponding negative steps observed during the particle binding phase. Indeed, QCM-based experiments using macroscopic electrodes exhibit rapid, extensive displacement.^27^ The absence of large positive steps (or large numbers of small steps corresponding to particles that are gradually released) indicates that the particles do not leave the host electrode. Repeating the experiment under different conditions such as 30 times faster flow rate and varied cleaning protocol (leading to different numbers of hosts) led to similar profiles.

We are thus led to two conclusions: (i) the entire ssDNA displacement and associated mass transport takes place within a few minutes and (ii) the detected negative steps during the target phase, which are only slightly smaller than the particle binding signature, are due to rearrangements of the particles when the DNA anchors are displaced. But how does a particle induce a large negative step on a neighboring electrode without leaving a large positive step on the host electrode? The mushroom-like geometry of the electrodes in our CMOS nanocapacitor arrays is sketched in Figure 4a. The electric field is stronger at the thin edges of the electrodes than at their center, a behavior that is analogous to the so-called edge effect for diffusive mass transport at ultramicroelecrodes.^31^ Thus, even though the projected area of a particle at the edge of an electrode is smaller than that at its center, a rearrangement from the center of the electrode toward its edge may not lead to a significant change in admittance for that electrode. It may, however, have a substantial influence on a neighboring electrode (Figure 4b). This ‘rolling’ process can only take place once the DNA anchors of the particle have been released, however (Figure 4c). Particles may become immobilized at the edges of the electrodes due to nonspecific interactions with the surface (the particles being now decorated with hybridized substrate and target DNA), or possibly through dielectrophoresis (see Section S9 for further discussion).

**Figure 4:**
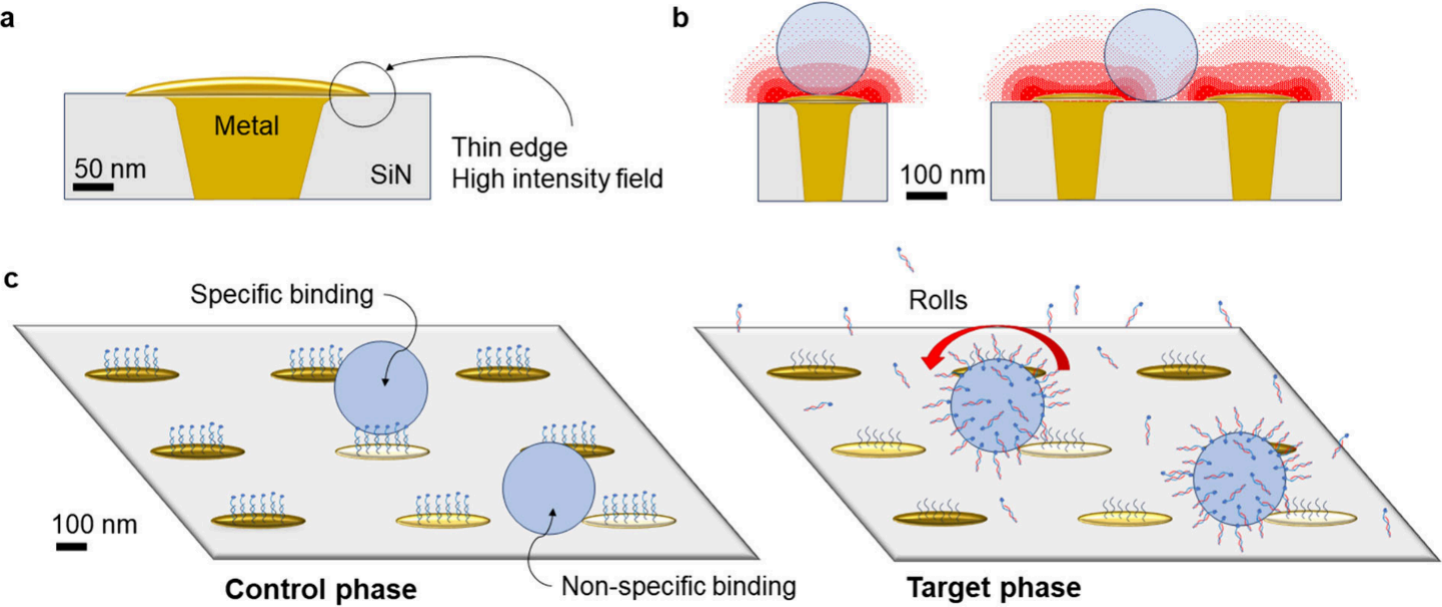
(a) A schematic of electrodes and their metal contacts, drawn to scale based on AFM and TEM data. (b) Sketch of the edge effect at an adjacent electrode (particle drawn to scale). Here the red region represents qualitatively the strength of the electric field. (c) The particle impacts on the hosts’ neighboring electrodes before (left) and after (right) DNA release.

Figure 5 (top row) shows a more quantitative analysis of the data of Figure 3 in the form of histograms of the step sizes. The corresponding data for a control experiment in which exposure to the incumbent and substrate were omitted are displayed in the bottom row. Figure 5a shows the distribution of detected step sizes during the particle binding phase. It exhibits a normal-like distribution peaking around −37 nS and is clearly separated from the small steps near zero. Figure 5b shows the distribution of the step sizes for the control experiment. Few large events are observed, peaking around −30 nS. The number of hosts decreased from 3182 in the initial experiment to 316 in the control experiment, indicating that most particles in the initial experiment were specifically bound. Based on these distributions we defined as hosts all electrodes that (i) exhibited a large step in the range −15 to −60 nS and (ii) did not exhibit subsequent unbinding (positive step larger than 0.7 of the binding step amplitude). Further details of the particle signature analysis can be found in Sections S7 and S8. As seen in Figure 2, particles bound near the edge of an electrode can cause signatures on neighboring electrodes. Consequently, signals from electrodes neighboring a host were also examined in a short time window (4.3 s or 20 frames) to avoid double counting a single binding event, as detailed in Section S6.

**Figure 5:**
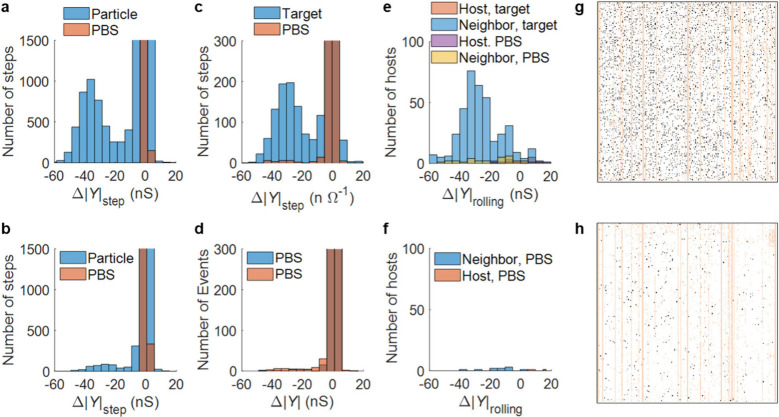
Binding–rolling analysis of a complete experiment. (a) Distribution of all the steps in the particle exposure phase, compared to that in the previous control phase. (b) Distribution of all the steps in the particle exposure phase compared to the previous PBS phase in a control experiment skipping incumbent and substrate exposure. (c) Distribution of all the steps in the target exposure phase, compared to that in the previous control phase. (d) Distribution of all the steps in the final PBS phase in the control experiment. (e) Distribution of the rolling factors of host and neighbor electrodes in the target exposure phase, compared to those in the previous control phase. (f) Distribution of rolling factor of host and neighbor electrodes in the final PBS phase in a control experiment. (g) Map of hosts (black pixels) in complete experiment. (h) Map of hosts in control experiment. In (g) and (h), electrodes shown in peach color were tagged as invalid.

The signals from the host electrodes were then analyzed during the target exposure phase. Figure 5c depicts the distribution of steps during this phase compared to the previous PBS exposure control phase. These distributions reflect the behavior observed in Figure 3: no large positive steps appear, while a large peak is observed around −30 nS (thus slightly smaller than in the particle phase and comparable to the control experiment). Only a few large steps occurred in the control experiment during the target phase, as seen in Figure 5d, so it can be concluded that the large peak around −30 nS in Figure 5c is a consequence of target exposure (see Figure S9a and the accompanying discussion for further analysis).

To further motivate the rolling mechanism, we defined the rolling factor for each host electrode as the sum of the sign-sensitive amplitude of all of the steps on the eight immediate neighboring electrodes during the target phase or the preceding PBS phase. By comparing this parameter during the control PBS phase and the target phase, we can evaluate its correlation with the presence of the target. Figure 5e presents the distribution of the rolling factor for host electrodes during both the target phase and the preceding control PBS phase, exhibiting a significant peak exclusively in the target phase. Figure 5f shows the same analysis for the control experiment, illustrating the rolling factor for nonspecifically bound particles. As expected, no such peak is observed in this case.

Figures 5g and 5h show maps of hosts on the electrode array in both complete and control experiments, again exhibiting a homogeneously scattered distribution.

These results provide insight into the dynamics of DNA-immobilized nanoparticles at nanoelectrodes. We found that the behavior of nanoparticles upon removal of their DNA tethers on our heterogeneous devices differs strongly from that observed on uniform metal surfaces, with the response being dominated by particle rearrangements on the surface rather than release into solution. To further transform this platform into a full-fledged stochastic biosensor, an important step will be to use smaller particles (diameter ≈ 50 nm). This will greatly decrease nonspecific adsorption, such that particles can return to solution instead of exhibiting rolling behavior; decrease the exclusion effect during the particle binding phase (potentially allowing for multiple particles per electrode); and decrease crosstalk between electrodes for particles binding near the edge of an electrode. The CMOS implementation of nanocapacitor arrays is essential here as it allows high-frequency operation, which is necessary for detecting particles at nanoelectrodes under physiological conditions, and provides the high level of parallelization required for stochastic detection.

## Supplementary Material



## Notes

Description of the measurement setup, materials, protocols, monitoring of electrode functionalization, data analysis, particle sizing and signature, average and single signals, tracking single particle dynamics, host interactions, hosts signature on neighbors, dielectrophoresis hypothesis, and experiments with 800 nm particles (PDF)
