# Supplementary material for: Digital Detection of DNA via Impedimetric Tracking of Probe Nanoparticles
Source: Nano Lett. 2025 Apr 22;25(25):9891–8. doi: 10.1021/acs.nanolett.4c05324 (PMC12203590; doi:10.1021/acs.nanolett.4c05324)
Supplement: Supplementary file 1 [file nl4c05324_si_001.pdf]

Supporting Information for
Digital Detection of DNA via Impedimetric Tracking of Probe Nanoparticles

Mohammad Saghafi, Suryasnata Tripathy, Taghi Moazzenzade, Jurriaan Huskens and Serge G. Lemay

Department of Molecules and Materials, Faculty of Science and Technology, University of Twente, Drienerlolaan 5, 7522 NB Enschede, The Netherlands.

Contents

S1. Measurement setup 1
S2. Materials 2
S3. Protocols 3
S4. Measurements 4
S5. Monitoring electrode functionalization 4
S6. Data analysis 5
S7. Particle sizing and signature 7
S8. Digital monitoring 8
S8.1. Average and single signals 8
S8.2. Tracking single particle dynamics 9
S8.3. Host interactions 9
S8.4. Hosts signature on neighbors 9
S9. Dielectrophoresis hypothesis 10
S10. Experiments with 800 nm particles 11
References 13

S1. Measurement setup

The experimental setup was composed of a programmable syringe pump (Pico Plus Elite, Harvard Apparatus), a customized socket, to provide reliable electrical contacts together with a sealed microfluidic chamber for the CMOS chip (designed and fabricated in collaboration with Yamaichi Electronics), a readout board, and custom software for data acquisition and monitoring [1]. Together, the socket, the chip and an EPDM gasket formed a microfluidic channel to which the nanoelectrodes were exposed, as shown in **Figure S1** (channel dimensions: 1000 × 220 × 200 μm LWH). The customized EPDM gasket, pre-cleaned in isopropyl alcohol (IPA) and treated with UV-Ozone (ProCleaner™ Plus, Bioforce Nanosciences) for 60 minutes, was used for ensuring a leak-proof interface. With the help of the syringe pump, controlled flow of liquids was achieved through the microchannel. The CMOS chip itself, as reported earlier [1, 2], was designed and post-CMOS modified by NXP Semiconductors. The gold/copper alloyed nanoelectrodes (~300 nm diameter) were arranged in submicron arrays, with 550 nm × 720 nm, 600 nm × 720 nm or 600 nm × 890 nm column/row pitch for different chip versions.

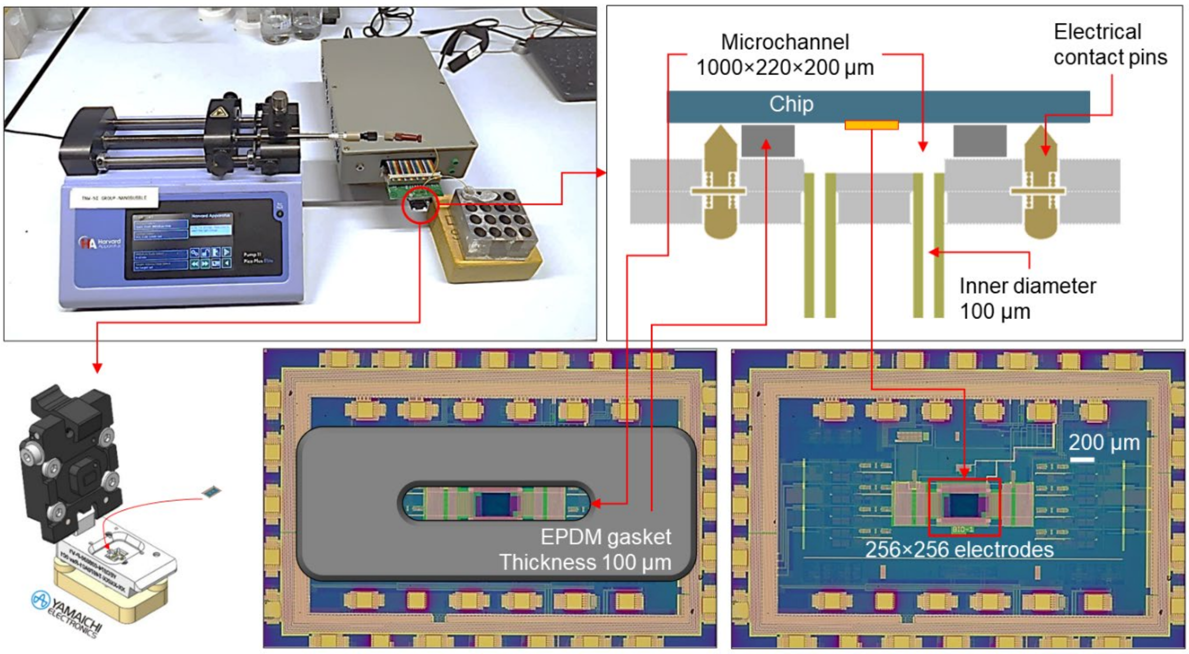

Figure S1. Experimental setup. Shown are the complete setup (top left), the chip (bottom right), the custom socket (bottom left), the chip with an aligned EPDM gasket (bottom center) and the microfluidic channel formed upon assembly (top right).

S2. Materials

The toehold-mediated strand displacement (TMSD) process is illustrated in Figure S2 for reference.

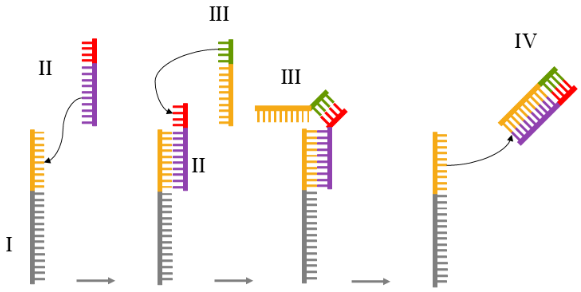

Figure S2. The toehold-mediated strand-displacement. Incumbent ssDNA (I) is hybridized with substrate ssDNA (II), then the target ssDNA (III) is attached to the toehold and competes with the incumbent. This ultimately leads to full displacement of the substrate from the incumbent, releasing the hybridized substrate-target strands (IV).

The incumbent DNA with 5'-thiol-C6 modifications (5′-SH-C6-AAAAAAATGTGTTGATGT-3′), the 5'-biotinylated substrate DNA (5′-TEG-AAAAACTTCCACTCACATCAACACA -3′) and the invader DNA (5′-TGTGTTGATGTGAGTGGAAG-3′) used in this work were purchased from Eurofins Genomic (Germany). The streptavidin coated polystyrene (SAV-PS) particles and the 6-Mercapto-1-hexanol (MCH) were purchased from Thermo Fisher Scientific and Sigma Aldrich, respectively. Chemicals including isopropanol (IPA), acetone, ammonium hydroxide (AMM), and phosphate buffered saline (PBS) were also purchased from Sigma Aldrich. All

chemicals were used as received. Milli-Q water from a Q-POD ultrapure water dispenser (Merck Millipore) was employed.

The incumbent DNA sequence contains 18 nucleotides, each approximately 0.34 nm long in B-form, resulting in a total nucleotide length of ~6.1 nm. The thiol-C6 spacer consists of 6 carbon atoms and a thiol group, contributing approximately 0.9 nm to the total length. Thus, the overall contour length of the incumbent is about 7.0 nm.

S3. Protocols

The measurement protocols are outlined in **Figure S3**. Prior to the experiments, the CMOS chip was cleaned in AMM, acetone and IPA in an ultrasonic bath and then subjected to air plasma treatment for 10 minutes. At the beginning of each experiment, the CMOS chip was subjected to a surface cleaning protocol, which involved its sequential exposure to IPA, milli-Q water, AMM 5% v, milli-Q water, and PBS, each for five minutes at a flow rate of 3 µL/min. These reagents served the following specific purposes: IPA facilitated surface wetting and prevented microbubble formation, AMM eliminated potential copper oxide from the nanoelectrodes, milli-Q water functioned as a rinsing medium, while PBS provided a baseline for probing the electrode's surface. The surface functionalization phase that followed was carried out at a flow rate of 0.1 µL/min, to allow the reactions of incumbent DNA (1 µM in PBS) and MCH (1 mM in PBS) at the nanoelectrodes to occur slowly and be distinguishable from the signature of the flow rate change. The incumbent DNA was immobilized on the nanoelectrode array using the gold-thiol self-assembly approach with a 45-minute incubation time. Nonspecifically bound polynucleotides were then washed away using PBS (5 min, flow rate 3 µL/min). In the next step, the electrode surface was blocked with MCH (90 min), followed by rinsing with PBS (5 min, flow rate 3 µL/min). Thereafter, biotinylated substrate DNA (1 µM in PBS) was introduced into the microchannel for 30 min at a flow rate of 0.1 µL/min for hybridizing with the incumbent. Subsequently, SAV-PS particles (1% w/v source, diluted 10 times in PBS) were captured on the hybridized duplex using biotin-streptavidin affinity binding for 90 minutes at a flow rate of 0.1 µL/min. Control PBS was followed by invader/target DNA (1 µM in PBS) were then introduced into the reaction chamber (each for 20 minutes, at a flow rate of 0.1 µL/min). At the end of the experiments, the microchannel was rinsed with milli-Q water and IPA. Notably, all liquid samples (excluding IPA) were degassed in advance to mitigate the risk of microbubble formation on the electrodes. Before switching the medium source, the flow was stopped for 1.5 minutes while the tube remained in the medium source. This precaution was taken to prevent the formation and entry of any bubbles into the tube. In addition, each phase that involved a 0.1 µL/min flow rate began with an initial flow rate of 3 µL/min for 45 seconds to account for the dead volume of the tube and ensuring that the analyte arrived at the electrodes without a significant delay. Electrical data were acquired continuously throughout these stages.

The selectivity of the assay was validated using a noncomplementary target DNA (5'-TATGAGGATCCTGATTGTGCTGTGT -3') using 800 nm particles. To test specificity, we used this dummy strand in place of the target DNA and compared the results to the plain PBS phase; both yielded the same outcomes. Therefore, for the 300 nm experiments, we used plain PBS exposure before the target DNA phase, ensuring the same duration for both control and experimental conditions. A negative control experiment was also conducted without the incumbent and substrate DNAs.

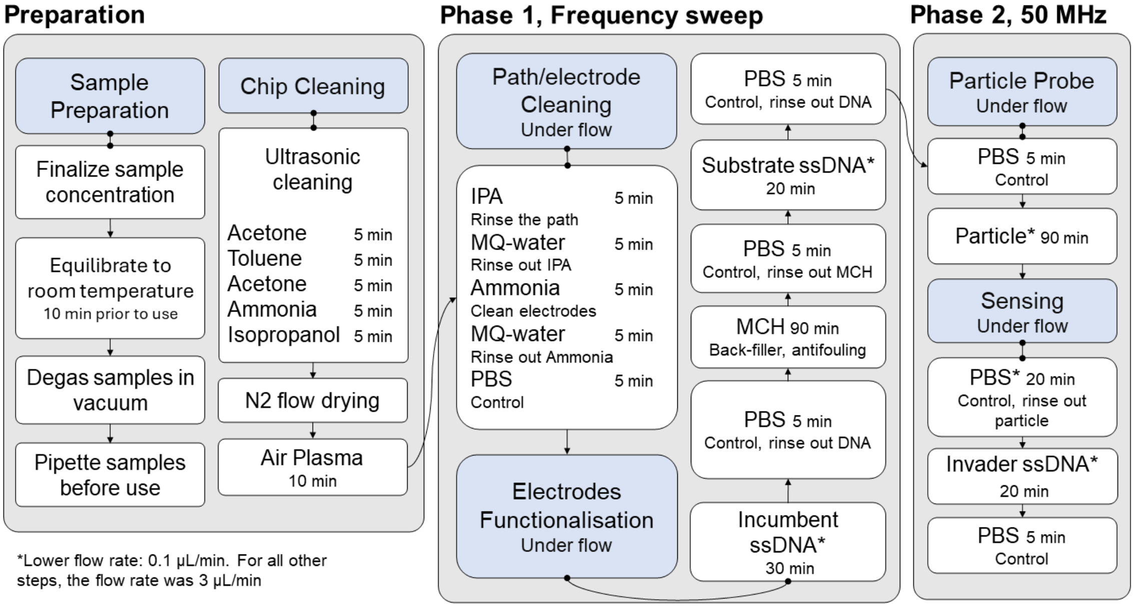

Figure S3. Experimental protocol flow chart.

S4. Measurements

We performed measurements with the CMOS-based nanocapacitor arrays in two distinct phases. Measurements in the first phase, that extended from surface cleaning up to the biotinylated substrate DNA hybridization step (as described in section S3), were recorded at 8 different frequencies from 1.6 to 50 megahertz (MHz). While the lower frequencies provided insights about events occurring remarkably close to the electrode's surface, i.e., within the electrochemical double layer (EDL), the higher frequencies allowed probing deeper into the bulk electrolyte. In contrast, in the second measurement phase that included the SAV-PS particle capture and the invader DNA-induced strand displacements, we used a single frequency of 50 MHz prioritizing faster monitoring frame rates.

S5. Monitoring electrode functionalization

In the first phase of the experiment, we monitored the functionalization of the electrodes at eight different frequencies. In general, lower frequencies probe changes occurring near the surface while higher frequencies probe the surrounding medium. The array underwent a rinsing step with PBS both before and after exposure to each molecule; we analyzed signal changes during molecular exposure by comparing the values obtained in PBS before and after the exposure for each functionalization step.

Figure S4a shows the average signal from all electrodes at eight frequencies during a surface functionalization experiment, followed by single-stranded DNA (ssDNA) displacement, in the absence of probe particles. The differences in signal caused by the incumbent DNA, MCH, and substrate phases are illustrated in **Figure S4b.** These graphs demonstrate that the changes detected over the substrate and MCH phases are more pronounced at low frequencies, which are sensitive to surface capacitance, and less pronounced at high frequencies, which are sensitive to bulk conductance.[3] Conversely, substrate binding occurring on top of these layers is less detectable at all frequencies. At low frequencies, sensitivity to the surface is impeded by the fully covered thiol-modified incumbent and MCH layers. At high frequencies, sensitivity to the bulk is limited because a single-stranded DNA

molecule is too small to significantly affect bulk conductivity. This limitation necessitates the use of a probe particle to significantly alter conductance by displacing a large enough volume of ions in the medium. Consequently, we utilized the highest frequency (50 MHz) for particle probing, which is most sensitive to their presence.

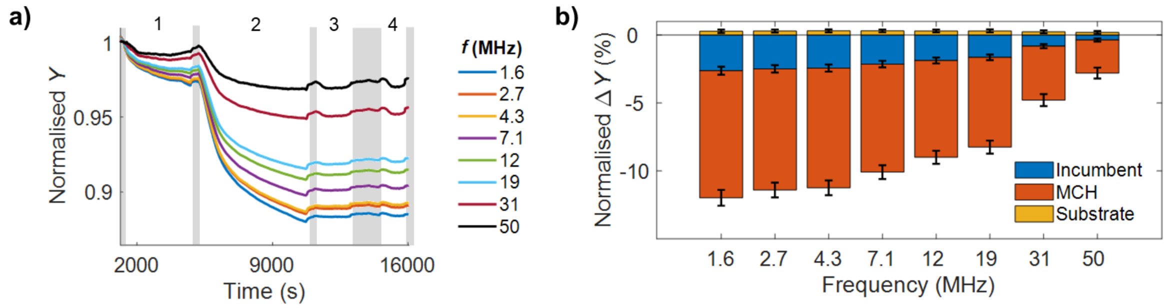

Figure S4. (a) Time trace of the average signal recorded across all valid electrodes at eight frequencies during a particle-free toehold ssDNA displacement experiment. Gray sections indicate PBS exposure, while the numbered sections represent: 1. Incumbent DNA, 2. MCH, 3. Substrate DNA, and 4. Invader/target DNA. Signals are normalized to the initial PBS phase average signal. (b) Average signal changes during the incumbent DNA, MCH, and substrate DNA phases, calculated by subtracting the PBS values before and after each corresponding phase. The error bars represent the standard deviation across the entire array.

S6. Data analysis

During the experiment, signals from 65,536 electrodes were recorded by a custom software and subsequently analyzed using MATLAB Version 9.14.0.2337262 (R2023a) Update 5. The data analysis procedure is summarized in **Figure S5.**

Initially, data of phase 1 were extracted and divided into time segments to avoid memory errors during analysis. The chip comprises of 256 columns of 256 electrodes, where each column is connected to a current read-out circuit that is transferred to eight analog to digital converters (ADC). As an initial step, we adjusted for variations between these ADC outputs. We assumed that the average output signals should be consistent across ADCs during a stable buffer exposure phase. Consequently, we adjusted each ADC's values to match the overall average of all ADCs. To achieve this, we used data from the initial PBS exposure during the first phase of the experiment. We first excluded columns with outlier average signals on the electrodes using the three-sigma rule. Next, we calculated the error for each ADC signal (each including 32 columns out of 256) as their deviation from the universal average in the stable phase. Finally, we corrected all signals of each ADC across all experiment frames using the corresponding error. For simplicity, we did not perform a full calibration as the steps we were focusing on were large enough that such precision was unnecessary[1].

The next step was to analyze the first phase of the experiment, which was recorded at eight frequencies from 1.6 to 50 MHz. This step reports on surface functionalization: cleaning the electrodes with AMM, thiolised incumbent DNA assembly, MCH assembly, and biotinylated substrate DNA assembly. We then identified invalid electrodes and defined a ‘Working Mask’. To define the Working Mask, first we monitored the electrodes’ signal changes between the first PBS exposure and the previous milli-Q water exposure. Electrodes showing outlier changes were excluded according to a three-sigma rule. Then the average and standard deviation of each electrode’s signal during the same PBS exposure phase were considered, and three-sigma outliers were again excluded from the Working Mask. Subsequently, the

average and standard deviation of signals from all valid electrodes were calculated at each frequency. Electrodes that deviated significantly from the total signal change in this phase (three-sigma rule) were removed from the Working Mask.

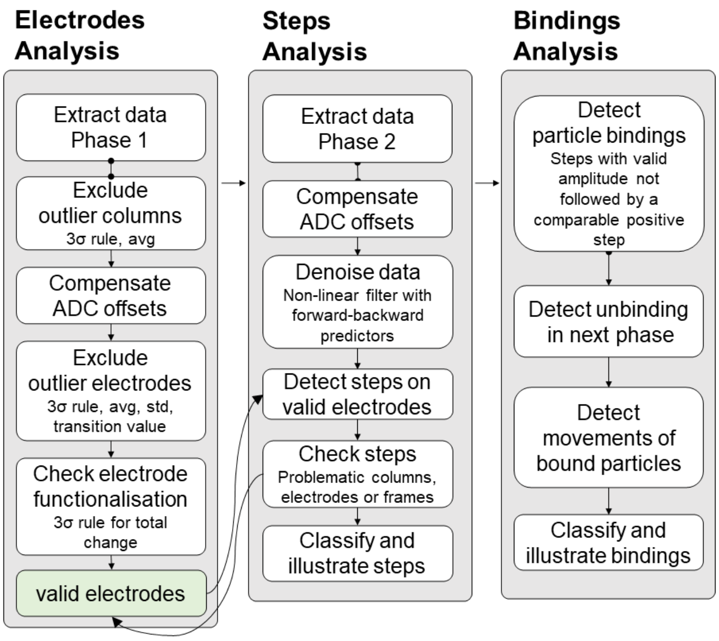

Figure S5. Data analysis flow chart.

Following the surface functionalization analysis and valid electrodes selection, we extracted the data of phase 2 measured at 50 MHz. We denoised the data and identified the steps to be considered for further analysis. Denoising was performed using a non-linear filter with forward-backward predictors so as to preserve the step structure[4]. For step detection, we used a difference window size of 100 and established a minimum threshold of 0.3 nanosiemens (nS): We shifted each signal 50 frames backward in time and subtracted it from the same signal shifted 50 frames forward in time. The result was then shifted 25 frames forward in time, which led to the representation of fluctuations and steps at each frame. Subsequently, any value less than 0.3 nS was set to zero to prevent the number of detected steps from becoming intractable due to noise. All remaining steps were then analyzed automatically.

We first analyzed the binding, unbinding, and rolling of particles on the electrodes. Our initial focus was on identifying negative steps within the desired amplitude range that were not followed by a comparable positive step of a comparable amplitude. Such cases were designated as 'host' electrodes. The neighboring electrodes of each host were then analyzed to avoid counting a binding on the edge of one electrode as a simultaneous binding at a neighboring electrode. Several cross-checks were implemented at this stage. Time steps containing an outlier number of steps were discounted on the basis that they could be caused by a sudden electrical or hydrodynamic fluctuation. Columns showing an anomalous number of steps were also discarded.

Signals from the hosts were subsequently examined during the control PBS phase and the invader DNA exposure phase to assess the particle dynamics, from unbinding to minor movements.

S7. Particle sizing and signature

We verified the size of the particles using Dynamic Light Scattering (DLS), as shown in **Figure S6a**. The measurement was carried out with a Malvern Zetasizer Nano ZS instrument equipped with a 633 nm laser set at an angle of 173°. Analysis was performed using software provided by the manufacturer (Zetasizer Software, Malvern). Subsequently the CMOS nanocapacitor arrays were exposed to particles nonspecifically bound to the electrodes. For this, we prepared an MCH layer on the electrodes as per the protocol described in experiment phase 1 outlined in section S3, excluding phases involving incumbent DNA and substrate DNA. After extracting and denoising the data, followed by step detection, the histogram of step sizes displayed a peak around –37 nS, extending to about 60 nS (**Figure S6b**). Consequently, we set the binding signature range for 300 nm particles from –15 to –60 nS. Applying the same methodology, we established a range of –15 to –70 nS as binding fingerprint for 800 nm particles.

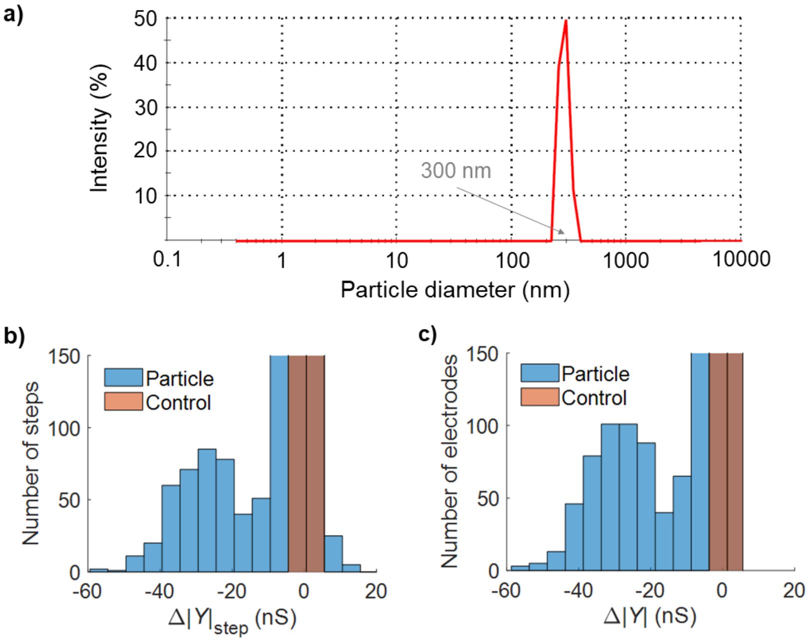

Figure S6. (a) DLS size analysis of SAV-PS particles. (b) Distribution of step sizes in the particle phase. There is a peak near –37nS, whereas the control (no particles) exhibits no such peak. (c) Distribution of the total signal change for each electrode during the particle phase compared to the control. The results follow the same pattern as (b), supporting the same conclusions.

S8. Digital monitoring

The strength of a nanosensor array lies in the fact that it can detect small events which would be undetectable at larger electrodes, while at the same time exhibiting an effective sensing surface comparable to that of a similarly sized microelectrode. The first point is illustrated in **Figure S7**, which compares the average response obtained from the complete sensor array to the response of individual nanoelectrodes. **Figure S8** instead shows data for 25 neighboring electrodes, which allows tracking particle movements.

S8.1. Average and single signals

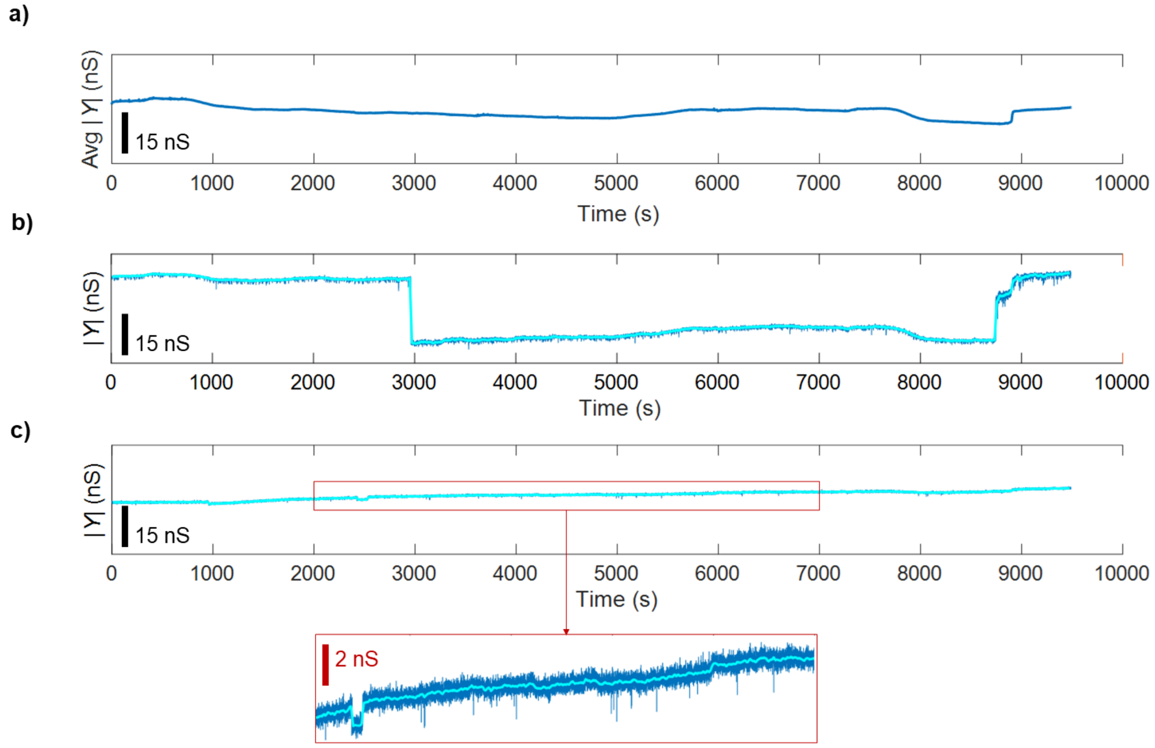

Figure S7. (a) The average signal of all valid electrodes (54,844 out of 65,536), and (b,c) the raw and denoised signals for two individual electrodes. The average in (a) only provides information about overall fluctuations such as temperature, while distinct negative and positive steps of ~25 nS are observed in panel b representing particle binding and unbinding. In addition to these large events, smaller events also occur on non-host electrodes as seen in panel c.

S8.2. Tracking single particle dynamics

Figure S8 shows the same data as Figure 2b with the addition of coordinate labels marking the position of specific step (the first two rows were removed to better focus on the region of interest). This illustrates that, while it is of course possible for different events to occur within a short time interval of each other, simultaneous signatures are normally only observed at electrodes that are immediate neighbors.

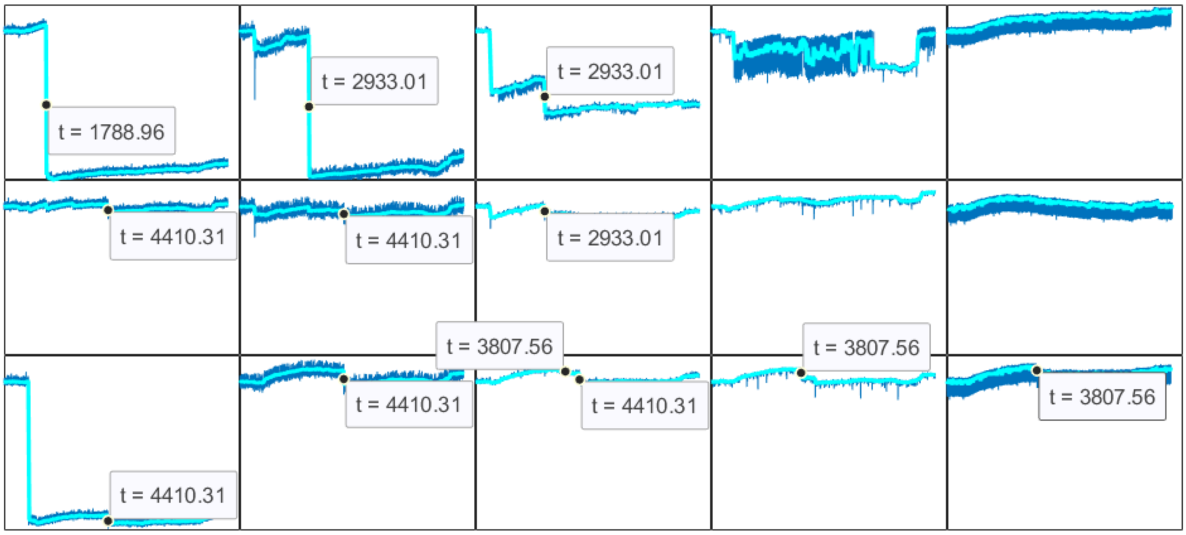

Figure S8. Particle dynamics tracking. These are the same data as Figure 2b with labels indicating the time of specific events in seconds.

S8.3. Host interactions

In the experiment of Figure 3 and Figure 5a,c,e,g, we detected 3,182 hosts out of 54,844 valid electrodes, indicating a 5.8% coverage. Assuming a random distribution, the expected average number of hosts in the eight neighboring electrodes of a typical host would be $8 \times 0.058 = 0.46$. We ensured that neighboring signals were not counted as additional bindings by filtering out recorded large simultaneous signals associated with host binding signals. Next, we assessed how many of these 3,182 hosts had another host in their vicinity. The result was zero in some data sets and only a few in others. We propose that the influence of a bound electrode on the surrounding flow, coupled with electrostatic repulsion among particles, are factors that reduced the expected average occupation of ~0.5 to zero.

S8.4. Hosts signature on neighbors

We compared the binding signatures on neighboring electrodes between specific and nonspecific binding scenarios to determine where particles are more likely to land in the presence or absence of specific binding. We defined the maximum binding signature of the electrodes neighboring the host and occurring at the same as the host binding. Higher parameters indicate larger binding signatures on neighboring electrodes, corresponding to particles that are further from the center of the host. The results (**Figure S9a**) show higher values for the control experiment, supporting, the conclusion that the attachment protocol leads to increased localization on the electrodes. Instead, nonspecific bindings are more likely to occur far from the center of electrodes.

Figure S9b illustrates the distribution of all detected steps in the target phase of the experiment, categorized into three classes: steps on host electrodes, steps on neighboring electrodes, and steps on other electrodes (referred to as ‘externals’). The steps on host

electrodes are relatively small (excluding the large step that defines the electrode as a host), whereas the steps on neighboring and external electrodes are more substantial. **Figure S9c** depicts the distribution for the control phase before target exposure, highlighting the rarity of large steps. Additionally, the analysis of an experiment excluding the incumbent and substrate phases (**Figure S9d**) yields results consistent with those of the control phase following the target phase.

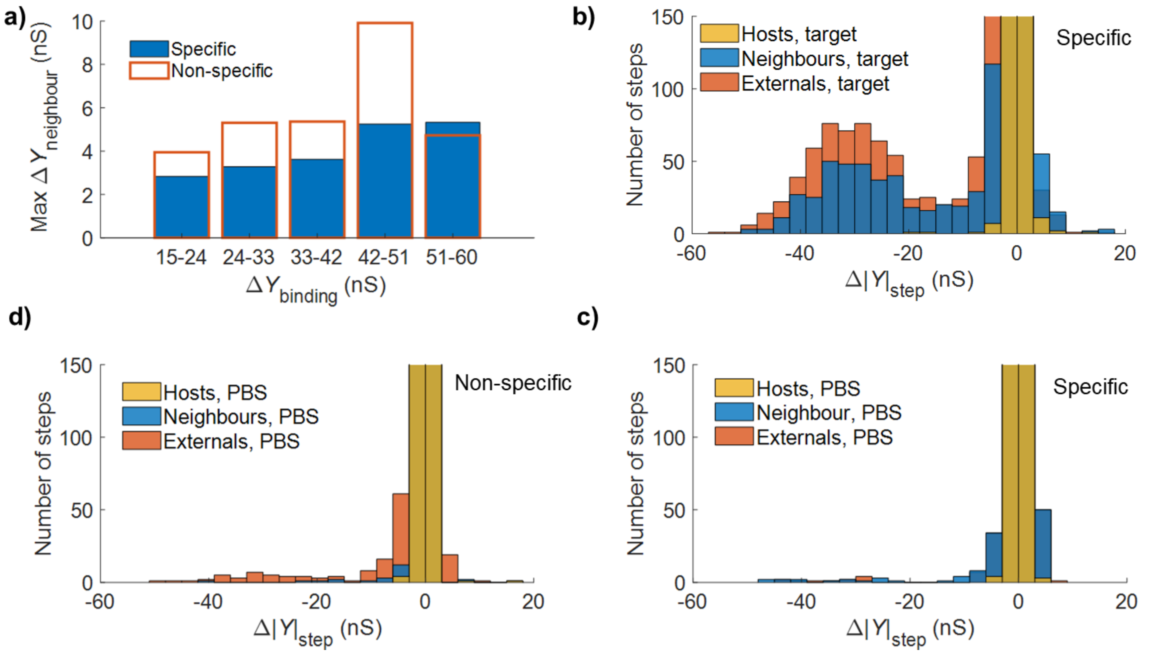

Figure S9. (a) Comparison of the maximum binding signature induced on neighboring electrodes between an experiment with electrodes functionalized with incumbent, MCH, and substrate, and a control experiment with electrodes covered only by MCH. Comparison of all the steps categorized into their corresponding electrode: host, neighbor or externals for (b) target exposure phase after buffer exposure for a complete experiment, (c) buffer exposure phase after particle binding before target exposure for a complete experiment, and (d) buffer exposure phase after particle binding of an experiment without incumbent and substrate treatment.

Given that particle rolling could be influenced by various factors and that many particles are nonspecifically staying on surface after ssDNA displacement, we cross-checked the large negative steps in the target phase. Electrodes that recorded steps with amplitudes larger than −15 nS during the target exposure phase were further examined. None of them were hosts, and around half of them were immediate neighbors to a host electrode (Figure S9b). For the other half, we could not establish a precise rationale to explain the experimental data. However, this does not impact the overall conclusion. It is possible that particles were released from some point along the line during target exposure. We observed very few bindings at neighboring electrodes, even during the particle exposure phase. Yet, during the target phase, there were a notable number of negative steps on neighboring electrodes with amplitudes exceeding 15 nS. This indicates that we are detecting particle rearrangements, rather than new particle bindings.

S9. Dielectrophoresis hypothesis

A consequence of the variation in electric field strength over the surface of the electrodes is that it can in principle induce dielectrophoresis (DEP). The DEP interaction with nanoparticles for the ionic strengths and frequencies employed here has been reported as negative (particles

repulsed from regions of high field strengths).[5] Our particles are however functionalized with DNA, creating a shell of counterions that increases surface conduction and favors positive DEP. Furthermore, the field gradients here occur on a length scale smaller than the particles themselves, whereas DEP is normally applied in the opposite regime of a smoothly varying field. Two additional factors are specific to the particularities of our experiments. First, our applied field is switched rather than simply harmonic, and DEP is a nonlinear effect such that higher forces may be generated. Secondly, our field distribution is highly heterogeneous because entire rows of electrodes are actuated and the neighboring rows serve as counter electrodes, with multiple rows contributing.[6] These differences may account for the discrepancy in frequency dependence. DEP would provide a mechanism by which particles would have a tendency to migrate toward and remain trapped near the edge of the electrodes upon release, however further extensive analysis would be required to prove or disprove this hypothesis.

S10. Experiments with 800 nm particles

We initially conducted experiments using 800 nm particles. However, since the particle diameter was more than twice that of an electrode's diameter, a particle's movements could affect many electrodes, leading to complex signatures. We therefore opted to use 300 nm particles, close to the electrodes' diameter. **Figure S10** shows the hosts map, a comparison of steps distributions in the PBS and following particle exposure phases, a comparison of steps distributions in the PBS and following target exposure phase. Top panels represent an experiment with the specific target and the bottom represent an experiment with the nonspecific target for control. Target exposure induces steps larger than PBS steps distribution (Figure S10b) but the nonspecific target doesn't (Figure S10f). Figure S10c indicates that most of these induced steps are hosts or around hosts, and are therefore related to the reorientation of particles on hosts induced by target displacement.

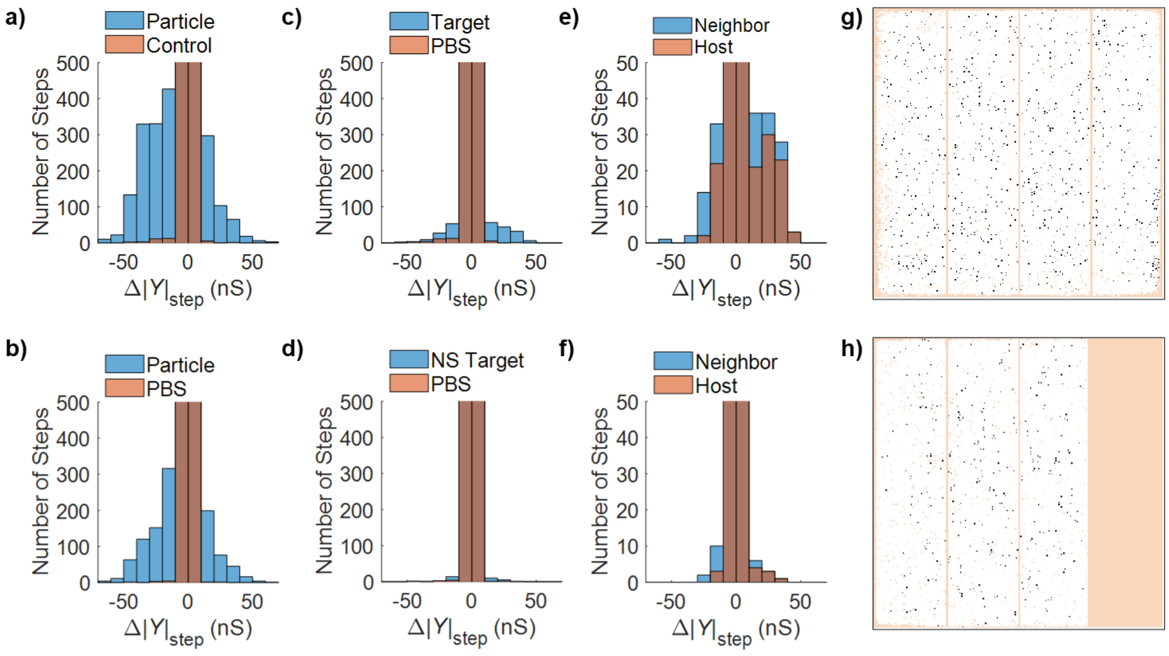

Figure S10. Statistical results of an experiment using 800 nm particle experiment. (a) Distribution of all steps during the particle phase compared to the preceding control phase for a complete experiment. (b) Distribution of all steps during the particle phase compared to the preceding control phase for a control experiment in which a nonspecific single-

stranded DNA is used instead of the target. (c) Distribution of all steps during the target phase compared to the preceding control phase. (d) Distribution of all steps during the nonspecific target exposure phase compared to the preceding control phase. (e-f) Distribution of rolling factors among hosts and neighbors compared the target phase and the preceding control phase for specific and nonspecific target experiments, respectively. (d-h) Map of the hosts for specific and nonspecific target experiments, respectively. Black pixels are hosts and pale peach pixels are invalid electrodes.

It is to be noted that the dynamics of 800 nm particles could be tracked with better clarity given their larger volume. **Figure S11** illustrates an example of this monitoring. Here we show a 3×3 grid (as opposed to a 5×5 grid in Figure 2) for two reasons: first, to ensure the figure remains clear and visible given its detailed content compared to Figure 2, and, second, because the relevant interactions were confined to this grid and not the outer layers. Our observations indicate that interactions do not significantly affect electrodes that are farther apart.

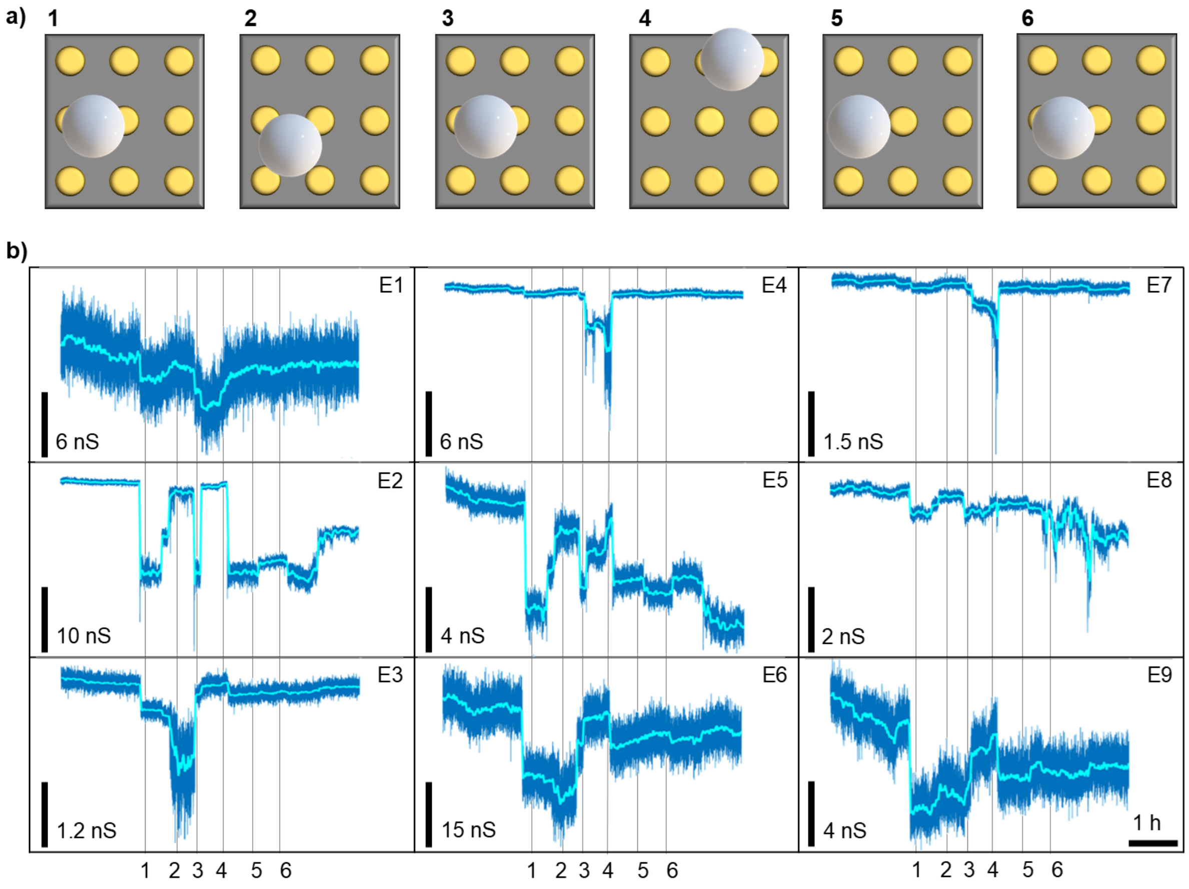

Figure S11. (a) Sketch of the inferred particle position on the electrodes at time points 1–6. The nine electrodes (golden disks) correspond for the nine signals in b. (b) Signals of nine neighboring electrodes hosting 800 nm particles. The axis numbers were removed for simplicity, and are replaced by scale bars for time and signal changes. Time points 1 to 6 are specified to illustrate the particle status. When the particle moves horizontally, the different electrodes show positive and negative changes at neighboring electrodes, depending on the direction of movement. At any given time, clock signals are applied to a vertical array, while the circuit readout is performed on a horizontal array.

References

(1) Widdershoven, F.; Cossettini, A.; Laborde, C.; Bandiziol, A.; van Swinderen, P. P.; Lemay, S. G.; Selmi, L. A CMOS Pixelated Nanocapacitor Biosensor Platform for High-Frequency Impedance Spectroscopy and Imaging. *IEEE Trans Biomed Circuits Syst* **2018**, *12* (6), 1369-1382, DOI: 10.1109/TBCAS.2018.2861558

(2) Lemay, S. G.; Laborde, C.; Renault, C.; Cossettini, A.; Selmi, L.; Widdershoven, F. P. High-Frequency Nanocapacitor Arrays: Concept, Recent Developments, and Outlook. *Accounts of Chemical Research* **2016**, *49* (10), 2355-2362, DOI: 10.1021/acs.accounts.6b00349 . Laborde, C.; Pittino, F.; Verhoeven, H. A.; Lemay, S. G.; Selmi, L.; Jongsma, M. A.; Widdershoven, F. P. Real-time imaging of microparticles and living cells with CMOS nanocapacitor arrays. *Nature Nanotechnology* **2015**, *10*, 791-795, DOI: 10.1038/nnano.2015.163

(3) Saghafi, M.; Chinnathambi, S.; Lemay, S. G. High-frequency phenomena and electrochemical impedance spectroscopy at nanoelectrodes. *Current Opinion in Colloid & Interface Science* **2023**, *63*, DOI: 10.1016/j.cocis.2022.101654 . Lazanas, A. C.; Prodromidis, M. I. Electrochemical Impedance Spectroscopy-A Tutorial. *ACS Meas Sci Au* **2023**, *3* (3), 162-193, DOI: 10.1021/acsmeasuresciau.2c00070

(4) Chung, S. H.; Kennedy, R. A. Forward-backward non-linear filtering technique for extracting small biological signals from noise. *Journal of Neuroscience Methods,* **1991**, *40*, 71-86, DOI: 10.1016/0165-0270(91)90118-J

(5) Bakewell, D. J.; Morgan, H. Measuring the frequency dependent polarizability of colloidal particles from dielectrophoretic collection data. *IEEE Transactions on Dielectrics and Electrical Insulation* **2001**, *8* (3), 566-571, DOI: 10.1109/94.933385 . Ermolina, I.; Morgan, H. The electrokinetic properties of latex particles: comparison of electrophoresis and dielectrophoresis. *J Colloid Interface Sci* **2005**, *285* (1), 419-428, DOI: 10.1016/j.jcis.2004.11.003

(6) Cossettini, A.; Laborde, C.; Brandalise, D.; Widdershoven, F.; Lemay, S. G.; Selmi, L. Space and Frequency Dependence of Nanocapacitor Array Sensors Response to Microparticles in Electrolyte. *IEEE Sensors Journal* **2021**, *21* (4), 4696-4704, DOI: 10.1109/JSEN.2020.3032712
